# Unraveling the Psychological Pathways Between Job Strain and Musculoskeletal Disorders: The Mediating Roles of Work‐Related Fatigue and Burnout Among Hospital Nurses

**DOI:** 10.1155/jonm/8819293

**Published:** 2025-12-28

**Authors:** Hsien-Hua Kuo, Cheng-Chieh Lan, Hsien-Wen Kuo, Ping-Yi Lin

**Affiliations:** ^1^ Department of Nursing, Taipei Hospital, Ministry of Health and Welfare, New Taipei City, Taiwan, mohw.gov.tw; ^2^ Institute of Environmental and Occupational Health Sciences, National Yang-Ming Chiao Tung University, Taipei City, Taiwan; ^3^ School of Public Health, National Defense Medical Center, Taipei City, Taiwan, ndmctsgh.edu.tw; ^4^ Department of Nursing, Hungkuang University, Taichung City, Taiwan, hk.edu.tw; ^5^ Department of Medical Research, China Medical University Hospital, Taichung City, Taiwan, cmu.edu.tw

**Keywords:** burnout, hospital nurses, job strain index, mediation analysis, musculoskeletal disorders, work fatigue

## Abstract

**Aim:**

This study examines the association between musculoskeletal disorders, job strain index, work‐related fatigue, and burnout among hospital nurses. Additionally, the mediating roles of work‐related fatigue and burnout in the correlation between job strain and musculoskeletal disorders were evaluated.

**Background:**

Musculoskeletal disorders often arise from high workloads and prolonged work hours among nurses. Despite extensive research, the definitive impact of psychological factors, work‐related fatigue, and burnout on musculoskeletal disorders remains inconclusive.

**Methods:**

A cross‐sectional study was conducted at a public hospital. The study adhered to STROBE guidelines and included 471 nurses who participated voluntarily and anonymously by completing a web‐based questionnaire. The data were collected from January to December 2022. The questionnaire covered demographics, job characteristics, the job control–demand–support model, work‐related fatigue, burnout, and other relevant factors. The mediating roles of work‐related fatigue and burnout in the relationship between job strain and musculoskeletal disorders were evaluated using Hayes Model 7.

**Results:**

Musculoskeletal disorders across nine body sites exhibited significant correlations with levels of job strain, work‐related fatigue, and burnout. Work‐related fatigue showed a significant association with burnout but did not directly associate with musculoskeletal disorders. Burnout, however, directly influenced the prevalence of musculoskeletal disorders. Notably, both work‐related fatigue and burnout served as mediators in the relationship between job strain and musculoskeletal disorders, with burnout being the primary contributor.

**Conclusion:**

Given that burnout is identified as the principal contributing factor to musculoskeletal disorders among nurses, it is recommended to implement empowerment initiatives within nursing departments. Prioritizing the reduction of work‐related fatigue and burnout through tailored prevention programs for nurses is essential.

**Implications for the Profession:**

The rising prevalence of musculoskeletal disorders calls for nurse managers to act promptly. They should implement intervention programs to reduce psychological stress and workload, enhance nursing management, and ensure an ergonomic workplace environment for nurses.

## 1. Introduction

Work‐related​ musculoskeletal disorders (MSDs) in healthcare professionals arise from a complex interplay of physical and psychosocial factors, including job strain, social support, and job dissatisfaction [1]. Traditionally, extensive research has focused on the risk factors associated with physical activities like manual handling and repetitive tasks, which can lead to discomfort, damage, or persistent pain in body structures, ultimately contributing to MSDs [2–4]. Nurses, in particular, experience high levels of emotional strain due to the intense demands of their work. A study found that nurses with symptoms of anxiety were more prone to MSDs, especially in the neck and shoulders, compared with their counterparts without such symptoms [5]. In a systematic review and meta‐analysis concerning hospital nurses and nursing aides, job‐related stress was associated with prevalent MSDs in multiple body regions (OR = 6.13). Additionally, low social support was associated with incident back pain (OR = 1.82) [2]. Despite low heterogeneity observed in most subsets of meta‐analysis, the roles of work‐related fatigue and burnout in the mechanism of MSDs were not distinctly assessed.

### 1.1. Job Strain in Nurses

Workers experiencing work‐related fatigue and burnout due to high time pressure, excessive demands, and insufficient social support may engage in repetitive movements and adopt awkward postures at work, potentially triggering or exacerbating MSDs [6]. When work‐related fatigue combines with job stress, burnout, and maladaptive pain responses, it becomes pivotal in the progression from acute to chronic MSDs. Studies have shown that levels of job strain among nurses, as derived from Karasek’s job demand–control (JDC) model, are associated with poor cardiovascular health, physical inactivity [7], and an elevated risk of neck, shoulder, and back disorders, particularly when job strain is coupled with perceived high physical exertion [8]. The JDC model provides a framework for understanding how job strain can lead to adverse health outcomes. However, while the JDC model effectively explains the role of job strain in various health conditions, it does not fully capture the psychological and emotional dimensions that might mediate the relationship between job strain and physical health outcomes, such as MSDs. Thus, we extend Karasek’s framework by incorporating two critical psychological mediators: work‐related burnout and fatigue.

### 1.2. Prevalence and Risk Factors of MSDs

A systematic review and meta‐analysis found that job strain significantly increased the risk of musculoskeletal pain, with findings indicating a rise of up to 62% [9]. Additionally, a study by Chung et al. [10] utilizing the Taiwan National Health Insurance Research Database from 2004 to 2010 reported an upward trend in MSDs among nurses. The annual incidences of MSDs and risk ratios increased from 28.35% in 2006 and 1.27 in 2004 to 33.65% and 1.46 in 2010, respectively. The authors attributed this trend to incorrect work‐related movements and psychological factors, though they did not explicitly consider the role of burnout. A comprehensive review by Davis and Kotowski [11] highlighted that among nurses, the prevalence of MSDs pain was highest in the lower back, followed by the shoulders and neck in hospital settings. Similarly, among Japanese nurses, MSDs in the previous year were most reported in the shoulder (71.9%), followed by the lower back (71.3%), neck (54.7%), and upper back (33.9%). The authors noted significant risk factors such as alcohol consumption, tobacco smoking, having children, heavy workload, and psychological factors contributing to the severity of MSDs.

### 1.3. The Roles of Work‐Related Fatigue and Burnout

The occurrence and prevalence of MSDs among healthcare employees stem from a multifactorial etiology, wherein physical demands and psychosocial risk factors exert independent and multiplicative effects [12, 13]. Prior research using structural equation modeling (SEM) identified psychosocial stressors and work–family conflict as mediators of MSDs and subsequent work‐related fatigue through stress and burnout (2017). Other studies have also demonstrated that fatigue may mediate the relationship between psychological strain and MSDs psychological factors and MSDs [14]. Despite previous findings, the precise causal pathway linking psychological factors, work‐related fatigue, burnout, and MSDs remains insufficiently clarified. Specifically, the direct and indirect effects of these variables on MSDs are still inconclusive. Most previous studies have primarily examined the direct effects of job strain on physical outcomes [15], often neglecting the mediating roles of psychological mechanisms like work‐related fatigue and burnout. Moreover, few studies have simultaneously examined these mediators across multiple body regions, and empirical evidence is notably lacking among Taiwanese hospital nurses.

Although numerous interventions have been proposed to alleviate work‐related fatigue, burnout, and MSDs, most focus narrowly on ergonomic or behavioral modifications, overlooking the psychological and organizational determinants that sustain these issues [16, 17]. Consequently, there is a pressing need for integrated models that capture both the direct and indirect pathways through which job strain contributes to MSDs, incorporating psychological mediators within a comprehensive framework.

Based on these research gaps, the present study seeks to empirically test a psychosocial pathway model linking job strain, work‐related fatigue, and burnout to MSDs among Taiwanese hospital nurses, thereby advancing theoretical understanding and informing more holistic prevention strategies. It is hypothesized that hospital nurses frequently experience high job strain, along with persistent work‐related fatigue and burnout, which subsequently increase the likelihood of MSDs. Therefore, the present study aims to (1) examine the associations among job strain, work‐related fatigue, burnout, and MSDs, (2) assess the mediating roles of work‐related fatigue and burnout in the relationship between job strain and MSDs among hospital nurses, and (3) provide empirical evidence to support the development of integrated psychosocial interventions tailored for hospital nurses, thereby advancing theoretical understanding and informing more holistic prevention strategies.

**Figure 1:**
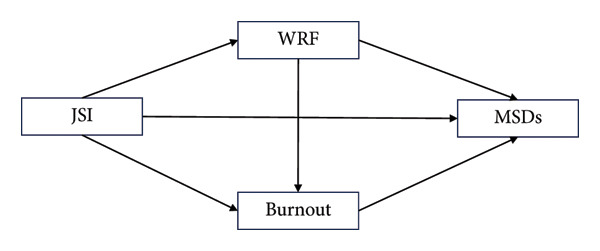
Schematic of the mediator of work‐related fatigue (WRF) and burnout for the association between job strain index (JSI) and musculoskeletal disorders (MSDs) using Hayes’s Model 6.

Accordingly, a theoretical framework was developed (Figure 1) to illustrate these hypothesized relationships, proposing that job strain indirectly affects MSDs through both work‐related fatigue and burnout. This model highlights the dual psychological pathways through which occupational stress may lead to adverse physical health outcomes in nursing contexts. The following hypotheses were constructed.

**Hypothesis 1.**

There is a significant positive association among job strain, work‐related fatigue, burnout, and MSDs.

According to Karasek’s JDC model, high job demands (JDs) combined with low control over one’s work (job strain) lead to increased stress and adverse health outcomes [18]. A high job strain index (JSI) may result in prolonged muscle tension, physical strain, and inadequate recovery, all heightening the risk of MSDs [19]. Furthermore, the model suggests that this stress contributes to mental and physical exhaustion (burnout and fatigue).

**Hypothesis 2.**

Work‐related fatigue mediates the association between JSI and MSDs such that higher job strain is associated with increased musculoskeletal symptoms through heightened levels of work‐related fatigue.

Fatigue is another critical factor impacted by job strain, especially among healthcare professionals who work extended hours or shifts. Persistent fatigue can lead to muscle tension and improper posture, heightening the risk of MSDs. By examining work‐related fatigue as a mediator, this study seeks to provide a more comprehensive understanding of how job strain translates into physical health issues. Research has shown that musculoskeletal symptom rates increase among employees experiencing poor work posture and fatigue, particularly among nurses [20].

**Hypothesis 3.**

Burnout mediates the association between JSI and MSDs, with higher job strain associated with increased musculoskeletal symptoms through increased burnout levels.

Burnout is a well‐recognized consequence of chronic job strain, especially in high‐stress occupations like nursing. The emotional exhaustion and depersonalization associated with burnout have been linked to both psychological and physical health outcomes. We hypothesize that burnout exacerbates the physical strain caused by JDs, contributing to a higher prevalence of MSDs. According to a study, burnout syndrome served as a significant mediator in the relationship between psychosocial risk factors and the intensity of MSDs, with burnout showing a strong association with increased MSDs intensity among hospital nurses [21].

## 2. Materials and Methods

### 2.1. Study Design and Participants

This study employed a cross‐sectional quantitative design using an anonymous web‐based questionnaire survey to investigate hospital nurses. Data were collected between January and December 2022 through convenience sampling. Before participation, all nurses were informed of the study objectives and provided informed consent. The minimum sample size was estimated using G‐Power Version 3.1.9.7∗ for multiple regression analysis. Assuming a medium effect size (*f* ^2^ = 0.10), *α* = 0.05, statistical power = 0.90, and nine predictors, the minimum required sample size was 207 participants. Accounting for an expected 20% attrition rate, at least 259 participants were needed to maintain adequate statistical power. Eligible participants met the following inclusion criteria: (1) registered nurses currently employed at a hospital, (2) having at least 3 months of work experience, (3) having provided informed consent, and (4) voluntarily completing the online questionnaire. Nursing students working in hospitals were excluded from participation.

A total of 471 hospital nurses from a governmental hospital in Northern Taiwan completed the survey, exceeding the minimum requirement and ensuring sufficient power for all planned regression and mediation analyses. Participants anonymously completed a structured questionnaire that comprised four sections: demographic characteristics, job characteristics, the Job Content Questionnaire (JCQ) [22], and the Nordic Musculoskeletal Questionnaire (NMQ), which is widely used to assess musculoskeletal symptoms in ergonomic evaluations. The response rate was 94%, and all participants received administrative support and an incentive of a 100 NTD gift card for their time and contribution.

### 2.2. Measurement

#### 2.2.1. Demographic Characteristics

The questionnaire collected information on participants’ sociodemographic characteristics and job‐related factors. Sociodemographic variables included age, education level, and marital status (single, married, and other). Work‐related variables included managerial position, work schedule (fixed and nonfixed), and job tenure.

#### 2.2.2. JCQ

Job strain was assessed using the JCQ, which evaluates psychological JDs, job control (JC), and social support. The JDC model, introduced by Karasek [23], is one of the most extensively studied frameworks in occupational stress research. In this study, a Chinese version of a screening tool based on Yeh’s [24] adaptation of the JDC model was used to identify high‐risk workplaces. JC comprised nine questions addressing job autonomy, characteristics, and work self‐determination. In contrast, JD encompassed eight items related to work pressure, exhaustion, heavy workload, limited time, and task ambiguity [24]. JC and JD were measured using a Likert scale: *strongly agree* (4 points), *agree* (3 points), *disagree* (2 points), and *strongly disagree* (1 point). The content validity of the questionnaire was reviewed by three specialists, including an occupational physician, epidemiologist, and occupational hygienist. The reliability of the JC and JD models, as measured by Cronbach’s *α*, were 0.83 and 0.82, respectively. JSI was determined by dividing JD scores by JC scores, representing perceived job‐related stress among nurses.

#### 2.2.3. Work‐Related Fatigue

Work‐related fatigue was assessed using a 5‐item Chinese version questionnaire developed by Cheng et al. [25], which has been widely used and validated in studies conducted within Chinese‐speaking populations. Responses were rated on a Likert scale with four options: “*always*” (3 points), “*usually*” (2 points), “*occasionally*” (1 point), and “*never*” (0 points). The decision to use this scale was based on its simplicity and proven reliability in assessing the frequency of fatigue symptoms in occupational settings, particularly in healthcare environments. In our study, the Cronbach’s *α* coefficient for work‐related fatigue was 0.92, indicating excellent internal consistency and supporting the robustness of this measurement tool.

#### 2.2.4. Burnout

Burnout levels were measured using the Chinese‐revised Copenhagen Burnout Inventory (C‐CBI) [24], a tool that has been adapted and validated for use in Chinese‐speaking populations. The C‐CBI assesses burnout experienced over the past year using seven items, each scored on a 4‐point Likert scale ranging from 0 (“*never*”) to 4 (“*always*”). The total score ranges from 0 to 28, with higher scores indicating higher levels of burnout. In the current study, the Cronbach’s *α* coefficient for burnout was 0.93, again demonstrating excellent internal consistency and the reliability of this instrument in measuring burnout among nurses. To better interpret the burnout data, we categorized participants into two groups: high burnout (≥ 15 points) and low burnout (≤ 14 points).

#### 2.2.5. NMQ

The NMQ was adapted from the previous questionnaire developed by the Taiwan’s Institute of Labor and Occupational Safety and Health (ILOSH, 2010), which itself was a revision of the questionnaire initially proposed by Kuorinka et al. in 1987 [26]. Previous studies have demonstrated the reliability and validity of the self‐reported NMQ, with reliability ranging from 77% to 100% and validity between 80% and 100% [27, 28]. Sensitivity and specificity of the NMQ in assessing pain in the last 7 days, compared with clinical examination, have been reported between 66%–92% and 71%–88%, respectively [29]. Each nurse self‐reported their experience of MSDs in the previous year using the NMQ tool, which categorizes pain into three levels: no soreness and pain (0 points), soreness and numbness (1 point), and pain (2 points) across nine body sites. The maximum score on the NMQ was 18, with higher scores indicating greater severity of MSDs. Prevalence rates of MSDs in each body site were determined based on self‐reported pain as well as professional diagnosis and treatment.

### 2.3. Statistical Analysis

All statistical analyses were performed using SPSS 24.0. Descriptive statistics are presented as the mean ± standard deviation (SD) for JSI, work‐related fatigue burnout, and MSDs, while demographic variables are expressed as percentages. The correlations between JSI, work‐related fatigue, burnout, and MSDs were assessed using Pearson correlation analyses. The association between high burnout groups and MSDs in nine body sites was calculated using univariate analysis, determining odds ratios. Hierarchical multiple regression analysis was employed to examine the levels of MSDs associated with demographics, job characteristics, JSI, work‐related fatigue, and burnout. The conceptual framework of MSDs guided the hypothesis testing regarding whether work‐related fatigue and burnout mediate the relationship between JSI and MSDs and whether JSI directly contributes to MSDs. To explore the mediating effects, a mediation model was constructed using Model 7 of the PROCESS macro for SPSS [30]. The bias‐corrected 95% confidence interval (CI) was computed with 5000 bootstrapping resamples to determine the significance of the mediation effects. Standardized regression coefficients depicted the extent of the study variables’ effects on MSDs, while the percentage of indirect effects of the two mediators was calculated relative to the total effect of JSI on MSDs. A significant mediating effect was inferred if the 95% CI of the indirect effect did not contain 0. Statistical significance was defined as a two‐tailed *p* value of < 0.05.

### 2.4. Ethical Considerations

The study was conducted according to the guidelines of the Declaration of Helsinki and approved by the local ethics committee of Taipei Hospital Ministry of Health and Welfare (Approval identification number: TH‐IRB‐0019‐0039). Participants were informed regarding the study’s purpose and objectives, emphasizing the importance of confidentiality, anonymity, and the option to withdraw voluntarily from participation at any stage of the research.

## 3. Results

The demographic characteristics of hospital nurses with and without MSDs are compared and detailed in Table 1. Significant differences were noted in age, marital status, and tenure between the two groups. Nurses experiencing MSDs were predominantly senior (54.9%), classified as having a marital status of “other” (76.3%), and had a job tenure ranging from 11 to 20 years (59.8%). However, there were no significant differences in education level, managerial position, or work schedule between the MSDs and non‐MSDs groups. Table 2 displays the correlations between burnout, work‐related fatigue, MSDs, and JSI. Significant correlations were found among the variables. Burnout exhibited significant correlations with work‐related fatigue (*r* = 0.768), MSDs (*r* = 0.246), and JSI (*r* = 0.281). Similarly, work‐related fatigue demonstrated significant correlations with MSDs (*r* = 0.152) and JSI (*r* = 0.281). Furthermore, JSI showed a positive correlation with MSDs (*r* = 0.126).

**Table 1:** Comparison of demographic characteristics for hospital nurses with and without MSDs.

	Without MSDs (*n* = 265) *n* (%)	With MSDs (*n* = 206) *n* (%)	*p*
Age (years)			< 0.001
≤ 30	120 (67.8)	57 (32.2)	
31–50	108 (50.9)	104 (49.1)	
≥ 51	37 (45.1)	45 (54.9)	
Education (years)			0.233
≤ 12	26 (45.6)	31 (54.4)	
13–15	82 (57.3)	61 (42.7)	
≥ 16–18	157 (57.9)	114 (42.1)	
Marital status			< 0.001
Single	147 (64.2)	82 (35.8)	
Married	109 (53.4)	95 (46.6)	
Other	9 (23.7)	29 (76.3)	
Managerial position			0.205
Yes	10 (43.5)	13 (56.5)	
No	255 (56.9)	193 (43.1)	
Work schedule			0.191
Fixed	175 (58.5)	124 (41.5)	
Nonfixed	90 (52.3)	82 (47.7)	
Job tenure (years)			0.002
≤ 3	99 (62.3)	60 (37.7)	
3–10	100 (61.7)	62 (38.3)	
11–20	41 (40.2)	61 (59.8)	
≥ 21	25 (52.1)	23 (47.9)	

Abbreviation: MSDs = musculoskeletal disorders.

**Table 2:** Correlation between work‐related fatigue, burnout, musculoskeletal disorders, and job strain index.

	Work‐related fatigue	Burnout	MSDs	JSI
Burnout	0.768^∗∗^			
MSDs	0.152^∗∗^	0.246^∗∗^		
JSI	0.281^∗∗^	0.382^∗∗^	0.126^∗∗^	
Mean ± S.D.	42.67 ± 19.39	12.64 ± 5.12	1.28 ± 1.87	0.58 ± 0.12

Abbreviations: JSI = job strain index, MSDs = musculoskeletal disorders, WRF = work‐related fatigue.

^∗^ *p* < 0.05.

^∗∗^ *p* < 0.01.

Table 3 presents the odds ratios (ORs) in the high burnout group associated with prevalent MSDs across nine sites. The prevalence rates of MSDs in the shoulder (27.8%), lower back (20.2%), neck (17.8%), and upper back (17.0%) among hospital nurses were notably high. Except for the hand/wrist site, MSDs in the remaining eight body sites showed significant associations with burnout. Nurses in the high burnout group consistently exhibited higher ORs for MSDs compared with those in the low burnout group. Notably, the highest ORs were observed for three disorder sites: 4.12 for the hip, 3.13 for the foot, and 2.59 for the upper back.

**Table 3:** Odds ratios (ORs) in the high burnout group associated with prevalent musculoskeletal disorders in nine body sites.

Sites	Low burnout group	High burnout group	OR (95% CI)
	*n* (%)	*n* (%)	
Neck	84 (17.8)	38 (25.5)	2.05^∗∗^ (1.27–3.33)
Shoulder	131 (27.8)	55 (36.9)	1.89^∗∗^ (1.24–2.88)
Upper back	80 (17.0)	40 (26.8)	2.59^∗∗^ (1.58–4.23)
Elbow	26 (5.5)	10 (6.7)	1.38 (0.61–3.11)
Low back	95 (20.2)	45 (30.2)	2.35^∗∗^ (1.48–3.74)
Hand/wrist	56 (11.9)	24 (16.1)	1.74 (0.99–3.08)
Hip	35 (7.4)	22 (14.8)	4.12^∗∗^ (2.02–8.43)
Ankle	43 (9.1)	20 (13.4)	2.02^∗^ (1.07–3.80)
Foot	54 (11.5)	30 (20.1)	3.13^∗∗^ (1.76–5.58)

Abbreviations: MSDs = musculoskeletal disorders.

^∗^ *p* < 0.05.

^∗∗^ *p* < 0.01.

Table 4 presents the correlation between the number of MSDs across nine body sites and levels of work‐related fatigue, burnout, and JSI, as determined through multivariate analysis after adjusting for covariates. The occurrences of MSDs across the nine body sites were categorized into four groups: 0, 1‐2, 3‐4, and > 5. The numbers of MSDs across these body sites exhibited significant correlations with levels of work‐related fatigue, burnout, and JSI, indicating a strong relationship.

**Table 4:** Numbers in nine body sites of musculoskeletal disorders correlated with work‐related fatigue, burnout, and JSI using multivariate analysis after adjusting for covariates.

	Numbers in nine body sites of MSDs				*p*
	0	1‐2	3‐4	> 5	
WRF	36.3 ± 2.5	39.0 ± 2.8	44.2 ± 3.9	46.5 ± 3.5	0.004
Burnout	11.2 ± 0.6	11.8 ± 0.7	13.1 ± 0.8	16.0 ± 0.9	< 0.001
JSI	0.54 ± 0.02	0.56 ± 0.02	0.56 ± 0.02	0.60 ± 0.02	0.023

*Note:* *p* values were calculated by test for trends.

Abbreviations: JSI = job strain index, MSDs = musculoskeletal disorders, WRF = work‐related fatigue.

Table 5 shows the mediation analysis of work‐related fatigue and burnout on the association between JSI and MSDs. While work‐related fatigue was not a mediator for the association between JSI and MSDs, its indirect effect accounted for 2%. Conversely, levels of burnout significantly mediated the association between JSI and MSDs, with its indirect effect accounting for 41.4%. Moreover, both work‐related fatigue and burnout acted as mediators in the association between JSI and MSDs, with their combined indirect effect accounting for 46%. However, the direct effect of JSI on MSDs was not significant, suggesting that its contribution may primarily be indirect through work‐related fatigue and burnout subsequently affecting MSDs.

**Table 5:** Mediation analysis of work‐related fatigue and burnout on the association between job strain index and musculoskeletal disorders.

Mediation effect	*B* (SE)	LLCI	ULCI	Indirect effect (%)
JSI ⟶ WRF ⟶ MSDs	0.083 (0.693)	−1.349	1.436	2.0
JSI ⟶ Burnout ⟶ MSDs	1.694^∗∗^ (0.600)	0.695	3.037	41.4
JSI ⟶ WRF ⟶ Burnout ⟶ MSDs	1.885^∗∗^ (0.647)	0.789	3.278	46.0

Abbreviations: JSI = job strain index, LLCI = lower limit confidence interval, MSDs = musculoskeletal disorders, ULCI = upper limit confidence interval, WRF = work‐related fatigue.

^∗^ *p* < 0.05.

^∗∗^ *p* < 0.01.

## 4. Discussion

In this study, work‐related MSDs predominantly affected hospital nurses, with the most prevalent occurrences reported in the shoulder (27.8%), lower back pain (LBP) (20.2%), neck (17.8%), and upper back (17.0%). While the overall prevalence of MSDs in this study was relatively lower compared with previous research, the pattern of pain distribution among nursing staff remained consistent with earlier findings. For instance, one study by Sorour and El‐Maksoud [31] reported high rates of MSDs in the neck (67.2%), shoulder (65.5%), and lower back (63.8%), while another study by Tinubu et al. [32] highlighted the prevalence of MSDs in the lower back (44.1%), neck (28.0%), and knees (22.4%). A meta‐analysis also revealed elevated rates of MSDs in nurses, particularly in the lower back (59.5%), neck (53.0%), and shoulders (46.8%). Nurses in developed countries tend to experience a higher prevalence of MSDs compared with those in developing countries [33]. These findings highlight consistent trends in the three major anatomical areas most affected by MSDs among nurses across studies. The nursing profession has been extensively studied worldwide, with a particular focus on LBP resulting from frequent patient lifting and manual handling. However, our study found the highest occurrence of MSDs in the shoulder, followed by LBP, neck, and upper back, which aligns with findings by Lin et al.​ [33] indicating prevalent MSD symptoms in the shoulder, neck, right wrist, and LBP. Although previous research has emphasized common risk factors for MSDs, such as prolonged static positions, patient lifting, and high patient loads [35], fewer studies have explored pain in the upper and lower extremities [11]. Moreover, the introduction of new computerized work scheduling processes among nursing staff has contributed to an increase in upper extremity MSDs. Changes in work schedules and job characteristics, including prolonged computer usage in nursing stations and frequent patient room visits, have intensified the risk of MSDs among nurses. Given that MSDs are a leading cause of workplace absenteeism among nurses and can adversely impact health‐related quality of life (HRQoL), work performance, and retention rates, nursing supervisors must prioritize intervention programs. These programs should focus on identifying key risk factors, particularly emphasizing the reduction of work‐related fatigue and burnout, and implementing ergonomic adjustments to workstation designs to ensure correct work posture and prevent the incidence of MSDs among nurses. Regarding psychosocial risk factors, a study found that participants experiencing MSDs in any body segment reported dissatisfaction with their occupation. They also highlighted issues such as inadequate staff cooperation, poor nurse–physician interactions, and insufficient support from immediate supervisors [36].

In this study, nurses in the high burnout group consistently exhibited higher ORs of experiencing MSDs across nine body sites compared with those in the low burnout group. Moreover, the numbers of MSDs across these body sites were significantly correlated with levels of JSI, work‐related fatigue, and burnout. Previous evidence has consistently shown a positive association between MSDs and high levels of JD and burnout involving nurses [31] and in underground coal miners [37]. Nurses experiencing MSDs in various body regions may have specific associations with working conditions in healthcare facilities, which encompass physical demands, performance expectations, frustrations, and overall workload. A study conducted in Japan by Yoshimoto et al. [38] among nursing personnel identified multidimensional factors, including individual, physical, psychological, and occupational aspects, significantly associated with disabling LBP. These factors included kinesiophobia, previous episodes of LBP, and insomnia, highlighting the complex interplay between various influences on MSDs among nurses. Interestingly, our study revealed that while JSI was significantly associated with work‐related fatigue and burnout, it was not directly associated with MSDs (*β* = 0.043). This indicates that symptoms of MSDs may be more strongly associated with work‐related fatigue and burnout than with perceived job stress alone. Supporting this, Bernal et al. [2], in a systematic review and meta‐analysis, identified a significant relationship between a high JSI and the prevalence of LBP, shoulder pain, knee pain, and pain at various anatomical sites.

Additionally, a high JSI was linked to the prevalence of MSDs across various anatomical sites, while low social support was associated with increased incidents of LBP. However, it is essential to recognize that psychological risk factors in the workplace should not overshadow the significant impact of work‐related fatigue and burnout on MSDs among nurses. This highlights the need for comprehensive interventions to address these complex, interrelated issues.

Burnout often emerges as a long‐term consequence of workplace stress, characterized by symptoms such as fatigue, diminished energy, depletion, debilitation, and reduced productivity [39]. Consequently, burnout has been linked to various negative outcomes and job disengagement, including job dissatisfaction, decreased organizational commitment, absenteeism, intentions to leave, and turnover. As nurses experience burnout, their enthusiasm for work diminishes, leading to lower productivity, diminished HRQoL, and increased susceptibility to injuries and illnesses. Furthermore, the negative effects of burnout, including stress symptoms such as headaches, chronic fatigue, muscle tension, and sleep disturbances, may result in deviations from standardized operating procedures in nursing practice, increasing the likelihood of MSDs in addition to mental health concerns. Sorour and El‐Maksoud [31] highlighted that JDs and the severity of LBP are positive independent predictors of burnout, whereas the level of JD is an independent predictor of the number of MSDs. This aligns with our findings indicating that work‐related fatigue was significantly associated with burnout (*β* = 0.718) but not directly associated with MSDs (*β* = 0.002). Burnout directly influenced the prevalence of MSDs (*β* = 0.288).

Given the high prevalence of work‐related fatigue and burnout among nurses providing direct medical care, numerous preventive strategies have been proposed, including enhancing communication skills, engaging in healthy activities, seeking social support, practicing gratitude, fostering teamwork [40], and implementing psychological interventions such as yoga, meditation, running clubs, and mindfulness training [41]. Emerging approaches grounded in self‐determination theory (SDT) have increasingly been acknowledged as protective factors against burnout. Evidence from SDT‐based research indicates that fulfilling the basic psychological needs of autonomy, competence, and relatedness serves as a psychological buffer against burnout by enhancing intrinsic motivation and sustaining well‐being. For instance, Playful Work Design (PWD), which integrates elements of fun and competition, has been shown to enhance employee engagement and proactive behaviors [42]. Similarly, supportive leadership has been demonstrated to strengthen employees’ intrinsic motivation and work engagement [43]. However, their theoretical and empirical relevance to musculoskeletal health in nursing populations remains limited. Additionally, managerial teams can mitigate the impact of work‐related fatigue and burnout, which is often attributed to repetitive movements and unhealthy postures at work, by empowering nurses with the necessary skills and support. Such empowerment initiatives may include ergonomic training, workload management, and continuous professional development, which together foster resilience and sustainable performance. In a broader organizational context, leadership styles and motivational mechanisms also play a pivotal role in shaping workplace behavior. Taken together, these findings suggest that management approaches combining intrinsic motivation enhancement and supportive leadership can effectively promote employee engagement, collaboration, and knowledge exchange, thereby contributing to the development of healthier, more dynamic, and creative workplace environments.

This study is the first in Taiwan to examine the mediating effects of work‐related fatigue and burnout on the relationship between job strain and MSDs across nine body regions. The findings provide a novel theoretical perspective by elucidating how the JSI may directly and indirectly influence MSDs through its effects on work‐related fatigue and burnout. This contributes not only to the local understanding of psychosocial pathways linking job strain to physical health outcomes but also offers a model that may be applicable in similar healthcare systems. Overall, the results advance theoretical and practical insights for developing integrated workplace health promotion strategies that address ergonomic, psychological, and organizational determinants of nurses’ well‐being.

## 5. Implications for Nursing Management

This study provides important guidance for nurse managers and healthcare institutions seeking to address job strain, work‐related fatigue, and burnout among hospital nurses. As the findings indicate that burnout, rather than job strain alone, is the primary driver of MSDs, managerial interventions should extend beyond ergonomic improvements to encompass strategies that strengthen nurses’ psychological resilience and recovery capacity.

Practical approaches include promoting shared decision‐making, enhancing nurses’ autonomy in scheduling, workload distribution, and task prioritization, and establishing regular assessments to identify individuals or units at higher risk. Nurse managers should also integrate mindfulness‐based programs, peer support systems, and stress management training into routine practice to cultivate coping skills and reduce emotional exhaustion. Furthermore, adequate staffing levels and structured opportunities for rest and feedback can help prevent chronic fatigue and burnout. By empowering nurses with greater control over their work environment and providing supportive resources, healthcare organizations can mitigate both psychological and physical strain. Such management strategies not only reduce burnout‐related MSDs but also contribute to a more sustainable, engaged, and healthy nursing workforce.

## 6. Limitations

This study has several limitations that should be acknowledged but also viewed as opportunities for future research. First, the cross‐sectional design and reliance on self‐reported data limit the ability to draw definitive causal inferences between job strain, work‐related fatigue, burnout, and MSDs. However, these findings provide an important foundation for hypothesis generation and offer preliminary evidence supporting potential psychosocial pathways. Second, the exclusion of nurses who may have left the workforce due to severe MSDs could introduce selection bias, potentially leading to an underestimation of the true associations observed. Third, the clinical severity of MSDs was not confirmed through physical examination, which may affect measurement precision. Nevertheless, these limitations highlight valuable directions for future research. Despite these limitations, the study highlights valuable directions for future investigation. Longitudinal and intervention‐based research is warranted to confirm the causal relationships identified here and to examine whether reducing burnout and fatigue can mitigate the onset or progression of MSDs over time. Incorporating objective clinical assessments alongside self‐reported measures would also strengthen the evidence base and provide a more comprehensive understanding of the complex interplay between psychological and physical health among nurses.

## 7. Conclusion

In conclusion, our results demonstrate a correlation between the number of MSDs across nine body sites and levels of JSI, work‐related fatigue, and burnout. Furthermore, this study contributes to the existing literature by incorporating psychological mediators into the JDC framework, providing new insights into the relationship between JSI and health outcomes among hospital nurses. Both work‐related fatigue and burnout mediate the association between JSI and MSDs, with burnout emerging as the primary contributor, accounting for 41% and 46% of the indirect effects. These findings underscore the critical role of psychological processes in translating occupational stressors into physical health problems. Addressing the increasing prevalence of MSDs among nurses, therefore, requires comprehensive interventions that integrate ergonomic, psychological, and organizational perspectives. Nurse managers should implement strategies aimed at reducing job strain, managing workload, and strengthening psychosocial support systems. At the organizational level, healthcare institutions should adopt policies that enhance JC, promote recovery from fatigue and burnout, and foster safe, sustainable, and health‐promoting work environments for nursing staff.

## Ethics Statement

The study was conducted according to the guidelines of the Declaration of Helsinki and approved by the local ethics committee of Taipei Hospital Ministry of Health and Welfare (Approval identification number: TH‐IRB‐0019‐0039).

## Disclosure

The manuscript titled “The Impact of Job Strain on Musculoskeletal Disorders in Hospital Nurses: The Mediating Role of Work‐Related Fatigue and Burnout” was presented at the 29th International Conference on Health Promoting Hospitals and Health Services, held from September 20–22, 2023, in Vienna. The content has since been revised and updated for submission to the *Journal of Nursing Management*. The authors confirm that this manuscript has not been published in full or in part elsewhere, except as an abstract in the conference. All authors gave final approval of the version to be published.

## Conflicts of Interest

The authors declare no conflicts of interest.

## Author Contributions

Hsien‐Hua Kuo, Cheng‐Chieh Lan, Hsien‐Wen Kuo, and Ping‐Yi Lin made substantial contributions to the conception and design, or acquisition of data, or analysis and interpretation of data and were involved in drafting the manuscript or revising it critically for important intellectual content. Each author has participated sufficiently in the work to take public responsibility for appropriate portions of the content and agreed to be accountable for all aspects of the work in ensuring that questions related to the accuracy or integrity of any part of the work are appropriately investigated and resolved. Hsien‐Wen Kuo and Ping‐Yi Lin contributed equally to this work.

## Funding

The authors received no funding for this research.

## Data Availability

The data that support the findings of this study are available on request from the corresponding author. The data are not publicly available due to privacy or ethical restrictions.
